# Acute and preventive pharmacological treatment of post-traumatic headache: a systematic review

**DOI:** 10.1186/s10194-019-1051-7

**Published:** 2019-10-21

**Authors:** Eigil Lindekilde Larsen, Håkan Ashina, Afrim Iljazi, Haidar Muhsen Al-Khazali, Kristoffer Seem, Messoud Ashina, Sait Ashina, Henrik Winther Schytz

**Affiliations:** 1 Danish Headache Center, Department of Neurology, Rigshospitalet Glostrup, Faculty of Health and Medical Sciences, University of Copenhagen, Copenhagen, Denmark; 2 Departments of Neurology and Anesthesia, Critical Care, and Pain Medicine, Beth Israel Deaconess Medical Center, Harvard Medical School, Boston, USA

**Keywords:** Head injury, Post-traumatic headache, Treatment, Traumatic brain injury, Concussion, Systematic review

## Abstract

**Background:**

Post-traumatic headache (PTH) is associated with considerable disability and reduced health-related quality of life. Despite the very high prevalence of PTH, there are no evidence-based guidelines for PTH treatment. Thus, we found it timely to provide a systematic review of the current literature on acute and preventive pharmacological treatment of PTH using PubMed and Embase databases.

**Findings:**

Included studies involved acute and preventive pharmacological treatment of headache attributed to traumatic injury to the head in adherence to the International Classification of Headache Disorders (ICHD) criteria. Of 1424 potentially relevant articles identified, 63 were retrieved for detailed evaluation and seven studies (one prospective and six retrospective) met the inclusion criteria. None of the seven included studies were randomized clinical trials (RCTs) or used a placebo-controlled study design.

**Conclusion:**

We found that there is a lack of high-quality evidence-based studies on the pharmacological treatment of PTH. Future studies are highly needed and must emphasize open-label studies with rigorous methodology or RCTs with a placebo-controlled design.

## Introduction

Post-traumatic headache (PTH) is a common secondary headache disorder [1] associated with considerable disability and reduced health-related quality of life [2]. Epidemiological data has shown a lifetime PTH prevalence of 4.7% in men and 2.4% in women [3], with migraine-like and tension-type-like headache being the most common headache phenotypes [4]. In addition, a substantial number of PTH patients experience disabling comorbidities such as symptoms of depression, anxiety, and sleep disturbances [5].

In the International Classification of Headache Disorders (ICHD) [6], PTH is defined by onset of headache within seven days following trauma or injury to the head and/or neck and is also further characterized as either acute (the first three months from headache onset) or persistent – the latter being if the headache persists beyond 3 months.

Despite a very high prevalence of PTH, there are no evidence-based guidelines for acute or preventive pharmacological treatment of PTH. The likelihood of patients receiving optimal treatment is therefore low, with a high risk of unnecessary treatment exposure. Patients with very frequent or daily headache following a trauma are also at risk of developing medication-overuse headache (MOH) [3].

Current pharmacological treatments for PTH are based on acute or preventive medications used for primary headache disorders [4, 5], since PTH often mimics a migraine-like or tension-type headache-like phenotype [6]. This approach lacks evidence and often results in poor treatment responses [7]. Furthermore, side effects to pharmacological treatment may conflict with PTH comorbidities such as depression and anxiety. Here, we review the current literature on acute and preventive treatment of PTH and provide a useful overview for clinicians. In addition, we address methodological limitations and provide recommendations for future research.

## Methods

### Data sources

We searched PubMed and Embase databases for articles on acute and prophylactic pharmacological PTH treatment. The search was performed on January 16, 2019 with the following search string: *(post traumatic headache OR post-traumatic headache OR posttraumatic headache OR post traumatic migraine OR post-traumatic migraine OR posttraumatic migraine OR post concussion headache OR post-concussion headache OR post concussion migraine OR post-concussion migraine) AND (treatment OR therapy)*. The search was performed from database inception until the date of the database search. The electronic database search was supplemented with manual searches for published, unpublished and ongoing randomized clinical trials (RCTs) in ClinicalTrials.gov. The search term was “*post-traumatic headache*”.

### Selection criteria

The search was limited to articles on human subjects published in English. We also reviewed the reference lists of relevant primary articles and reviews to identify studies that were missed in the search process. All eligible studies were screened in accordance with the Preferred Reporting Items for Systematic Reviews and Meta-Analyses (PRISMA) reporting guidelines.

### Study inclusion and data extraction

**Fig. 1:**
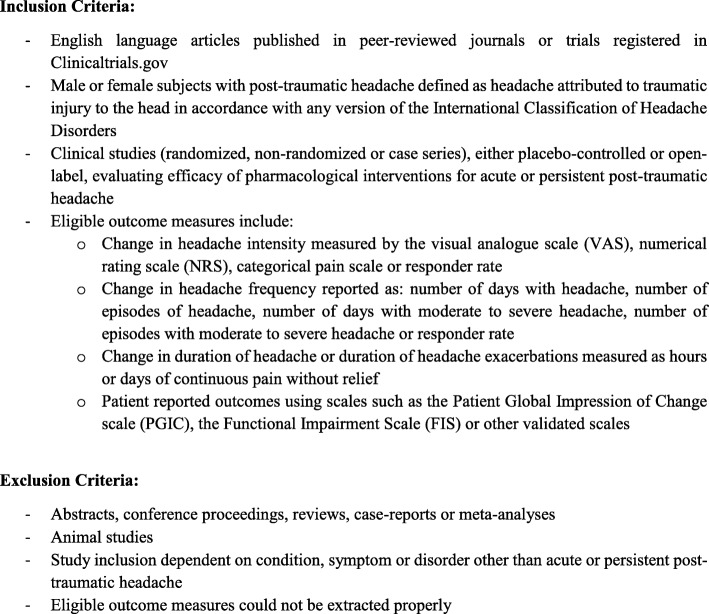
Eligibility criteria

Inclusion and exclusion criteria are presented in Fig. 1. We only included studies in which the subjects fulfilled the diagnostic criteria for *headache attributed to traumatic injury to the head* in accordance with any version of the International Classification of Headache Disorders (ICHD) [8]. One investigator (E.L.L) screened all articles by title and abstract. Following this, two investigators (E.L.L and A.I.) performed a full-text screening and determined which articles should be included. Another investigator (H.M.A) subsequently reassessed all the included articles. Final inclusion was decided by consensus between the three investigators (E.L.L, A. I and H.M.A). If consensus was not reached, one independent investigator (H.A) was available to provide advice. For each study, two investigators (E.L.L and A.I) recorded data on study design, assessment methods, inclusion criteria, exclusion criteria, age, gender, total number of subjects, headache phenotype, outcome measures and other data relevant for the scope of this review.

## Results

**Fig. 2:**
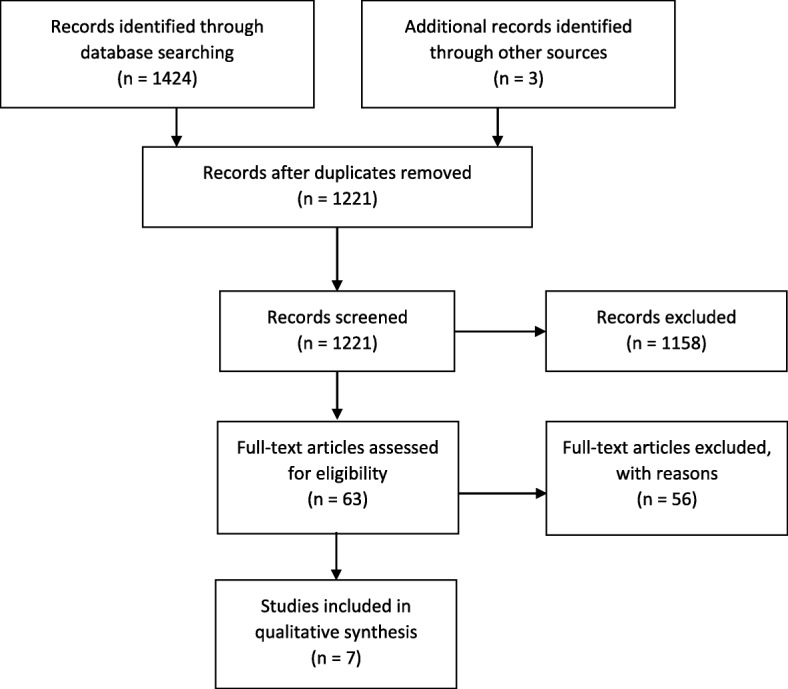
Flowchart

The database search resulted in a total of 1218 search hits (Fig. 2). Three additional articles were identified through a manual search of the reference lists of relevant primary articles and reviews. A total of 1221 articles were screened by title and abstract of which 63 articles were retrieved and assessed for eligibility in the present study. Seven articles met the inclusion criteria and were included in the qualitative synthesis [9–15]. Four studies [9–12] reported on acute PTH treatment, two studies [13, 14] on preventive PTH treatment and one study [15] on both acute and preventive PTH treatment. The studies were very heterogeneous in terms of the study populations assessed. Thus, three of the seven studies consisted of pediatric populations [9, 10, 14], one study was in children or adolescents [12] and one was in a population with both adults and children [13]. The rest consisted of a study on military personnel [15], and one study in an adult population [11]. None of the seven included studies were RCTs or included a placebo-controlled study design. Six studies collected data retrospectively [9, 10, 12–15] while one study acquired data prospectively [11].

Here, we present data focusing first on acute pharmacological treatment of PTH and then on preventive pharmacological treatment of PTH. Table 1 presents study characteristics for each of the seven included studies.

**Table 1:** Overview of included studies

Source	Patient Population, Baseline	Study Design	TBI Severity	Intervention	Follow-up	Eligible Outcome Measures	Major Findings
Chan et al. (2015) [12]	254 children and adolescents, 124 M^a^, 130 F^a^, mean age 13.8 years	Single-center, ED, tertiary children’s hospital, retrospective	mTBI	Acute Pharmacological Treatment. IV therapies included one of the following four options: - ketorolac only (*n* = 55) - ketorolac and metoclopramide / prochlorperazine (*n* = 132) - metoclopramide or prochlorperazine (*n* = 30) - ondansetron only (*n* = 37) The dosages used were not reported. Thirty-seven percent of the subjects were pretreated with either acetaminophen or ibuprofen.	None	Treatment success defined as ≥50% reduction in pain intensity as measured on a numeric rating scale from 0 to 10.	Treatment success: - Ketorolac only: 80% - Ketorolac plus metoclopramide or prochlorperazine: 89% - Metoclopramide or prochlorperazine only: 93% - Ondansetron only: 78%
Dubrovsky et al. (2014) [10]	28 children, 6 M^a^, 22 F^a^, mean age 14.6 years	Single-center, tertiary referral center, retrospective	mTBI	Acute Pharmacological Treatment. - GON block (2% lidocaine with epinephrine) - Peripheral nerve blocks of the lesser occipital nerve and supraorbital nerve (only a subgroup of patients)	Follow-up was conducted using a patient satisfaction survey. Five patients lost to follow-up. The exact time from intervention to follow-up could not be extracted properly.	Good therapeutic effect defined as headache relief lasting longer than 24 h or requested repeat blocks.	- 93% with good therapeutic effect - 71% reported complete headache resolution immediately following the intervention - At the follow-up assessment (82% follow-up response rate), 26% of patients reported that peripheral nerve blocks had *cured* their headache
Erickson (2011) [15]	100 military personnel, 99 M^a^, 1 F^a^, mean age 28.7 years	Single-center, clinic-based, retrospective	mTBI	Acute Pharmacological Treatment. - Triptans (n = 73) - Non-triptans (NSAIDs, acetaminophen, opioids and combination drugs^b^, *n* = 33) The dosages were not reported. Notably, 23% used more than one abortive medication. Preventive Pharmacological Treatment. One of the following therapies were prescribed: - Tricyclic antidepressants (amitriptyline or nortriptyline, *n* = 48) 25–50 mg/day - Topiramate 100 mg/day (*n* = 29) - Propranolol LA 80 mg/day (*n* = 18) - Valproate extended release 500 mg/day (*n* = 5)	3 months after treatment start, none lost to follow-up. At 3 months post-baseline, 66 of 100 subjects (34% medication discontinuation rate) were still taking the prophylactic treatment that was prescribed at baseline.	Acute Pharmacological Treatment. - Headache relief within two hours after intake (not further specified Preventive Pharmacological Treatment. - Headache frequency defined as number of days in the previous month with a headache lasting > 30 min - Headache-related disability as determined by MIDAS	Acute Pharmacological Treatment. - Triptans (*n* = 73 and a 70% responder rate) were more effective compared to non-triptans (n = 33 and a 42% responder rate) in terms of headache relief within two hours after intake (*P* = 0.01) Preventive Pharmacological Treatment. - The decrease in headache frequency was significant for subjects treated with topiramate (n = 29, *P* = 0.02), but not for any other of the prescribed prophylactic treatments - 57% overall decrease in headache-related disability among all subjects as measured by MIDAS
Friedman et al. (2018) [11]	21 adults, 5 M, 16 F, mean age 45 years	Single-center, ED, prospective	NS	Acute Pharmacological Treatment. IV metoclopramide 20 mg + dephenhydramine 25 mg.	48 h + 7 days, 2 patients lost to follow-up.	“Sustained headache relief for 48 h”, defined as mild headache or no headache sustained for 48 h since ED discharge without use of rescue medication.	- At 48 h since ED discharge, 63% reported sustained headache relief for 48 h, while 37% continued to experience moderate to severe headaches - At the 1-week follow-up, 53% reported no or rare headache occurrence
Kuczynski et al. (2013) [14]	44 children, 15 M, 29 F, mean age 14.1 years	Single-center, clinic-based, retrospective	mTBI	Preventive Pharmacological Treatment. The list of prophylactic treatments used included: - Amitriptyline 5 mg to 1 mg/kg (*n* = 18) - Topiramate 12.5–200 mg/day (*n* = 6)^a^ - Melatonin 3–10 mg/day (*n* = 12) - Nortriptyline (*n* = 9)^a^ and flunarizine (*n* = 8)^a^ doses were not specified - 17 subjects received more than one treatment i.e. physical therapy and biofeedback therapy	The mean follow-up rate was every 5.5 weeks until headache symptoms had resolved. None lost to follow-up.	Treatment success defined as ≥50% reduction in headache frequency and whether prophylactic treatments were continued for 3 months after headache resolution and subsequently gradually discontinued.	Treatment success: - Amitriptyline: 72% - Melatonin: 75%
Seeger et al. (2015) [9]	15 children, 5 M, 10 F, mean age 15.5 years	Single-center, clinic-based, retrospective	mTBI	Acute Pharmacological Treatment. Administration of GON block was done with 2,5 mL 2% lidocaine (50 mg) + 0.5 ml methylprednisolone acetate (20 mg) or 2.5 ml triamcinolone (25 mg).	Follow-up assessment was conducted at a mean of 5.6 months after treatment start, 1 patient lost to follow-up.	Full treatment response defined as ≥50% reduction in headache frequency.	Full treatment response: - 64% (9 of 14 patients) - Mean headache frequency was reduced from 26 days per month to 18 days per month
Cushman et al. (2019) [13]	277 children and adults, 139 M^a^, 138 F^a^, mean age 23.0 years^a^	Single-center, academic sports medicine practice, retrospective	mTBI	Preventive Pharmacological Treatment. Patients were classified into three groups: - No medication (n = 123) - Amitriptyline (median dose: 20 mg, *n* = 94) - Gabapentin (median dose: 900 mg, *n* = 60)	Follow-up data was collected over 1 year after treatment start. Study inclusion was dependent on at least one follow-up assessment.	Self-reported headache score, ranging from 0 to 6 (0 = no symptoms, 5–6 = severe symptoms).	- In both medication groups (gabapentin and amitriptyline), headache scores improved over time - However, headache scores improved similarly in the no medication group

*M* males, *F* females, *mTBI* mild traumatic brain injury, *NS* not specified, *ED* emergency department, *PTH* post-traumatic headache, *IV* intravenous, *GON* greater occipital nerve, *NSAIDs* nonsteroidal anti-inflammatory drugs

^a^Data has been calculated based on data provided in the studies

^b^Includes Excedrin, Cafergot, and Midrin

### Acute pharmacological treatment of post-traumatic headache

Five studies [9–12, 15] investigated acute pharmacological PTH treatment. The list of acute medications included common analgesics such as ketorolac [12], unspecified non-steroidal anti-inflammatory drugs (NSAIDs) [15], acetaminophen [15] and combination drugs (Excedrin, Cafergot and Midrin) [15]. Specific analgesics used were lidocaine [9, 10], triptans [15] and opioids [15]. Antiemetics used were ondansetron [12], metoclopramide [11, 12] and prochlorperazine [12]. Other drugs used were diphenhydramine [11], methylprednisolone acetate [9] and triamcinolone [9].

A retrospective cross-sectional emergency department (ED) based study investigated the treatment response of intravenous acute migraine therapies in an adolescent (aged 8–21 years) group consisting of 254 subjects [12]. All participants presented to the ED within 14 days following mild traumatic brain injury (mTBI). The subjects received one of the following four intravenous (IV) acute treatment options: 1) ketorolac only (*n* = 55), 2) ketorolac and metoclopramide/prochlorperazine (*n* = 132), 3) metoclopramide or prochlorperazine (*n* = 30) and 4) ondansetron only (*n* = 37). The dosages used were not reported. In addition, 95% of the subjects were discharged from the ED and 37% received pre-treatment with oral analgesics (acetaminophen or ibuprofen) in the ED. The primary outcome was ≥50% reduction in headache intensity score during the ED visit as measured on a numeric rating scale (NRS) from 0 to 10. The overall treatment response was 87% with approximately half of the subjects reporting complete resolution of headache. The treatment response of each of the four drug combinations is described in Table 1.

In a retrospective case series study, 28 children with PTH (aged 10–17 years) reported tenderness over the greater occipital nerve (GON) following palpation [10]. The patients were then treated with peripheral nerve blocks of the scalp, including GON blocks, lesser occipital nerve blocks and supraorbital nerve blocks. The blocks contained 2% lidocaine with epinephrine. The primary outcome was to examine the proportion of patients with *good effect* (pain relief > 24 h and/or repeat blocks requested), *partial effect* (pain relief < 24 h and no subsequent request for repeat blocks) or *no effect* (poor or no pain relief following intervention). Pain relief was defined as < 3/10 pain intensity on a NRS from 0 to 10. The first peripheral nerve blocks were conducted at a mean of 70 days since the mTBI. Fifty-seven percent of the subjects had a history of multiple concussions, while 70% had a history of headache prior to the mTBI. Ninety-three percent of the subjects reported good therapeutic effect, while the rest reported partial effect. In addition, 71% of the subjects reported complete headache resolution following the intervention. A patient satisfaction survey was conducted post-intervention (82% follow-up response rate) with 26% of subjects reporting that peripheral nerve blocks had *cured* their headache. The time interval from treatment start to survey response was not further specified. Moreover, the authors reported that 82% received intravenous metoclopramide treatment prior to the first treatment with peripheral nerve blocks. Following metoclopramide treatment, 32% of subjects reported complete immediate headache resolution while 44% achieved partial and 24% no headache relief, respectively. Intravenous metoclopramide treatment was conducted at a mean of 39 days since the mTBI. The authors did not report the dosages used for intravenous metoclopramide treatment.

In another case series, Seeger et al. [9] performed a retrospective chart review in 15 children (mean age: 15; range 13–17) with PTH attributed to mTBI, who were treated with GON blocks. Follow-up data was obtained from 14 of 15 subjects at 6 months after the intervention. The time range from injury to intervention was 1–12 months with 11 subjects reporting persistent PTH (duration > 3 months). The primary outcome was “*full response*” defined as ≥50% reduction in headache frequency or “*partial response*” defined as reduction in headache frequency less than 50%. Sixty-four percent of the subjects reported “full response” at the follow-up assessment, while one subject reported “partial response”. Mean headache frequency was reduced from 26 ± 7 days per month pre-mTBI to 18 ± 12 days per month post-mTBI (*P = 0.014*).

A prospective ED-based open-label study investigated headache relief in 21 patients treated with intravenous metoclopramide 20 mg and diphenhydramine 25 mg after acute PTH attributed to TBI [11]. All patients reported TBI within the past 10 days at the time of intervention, and 90% provided data at both the 48 h and one-week follow-up. The primary outcome was “*sustained headache relief for 48 h*”, defined as mild headache or no sustained headache for 48 h since ED discharge without use of rescue medication. At 48 h since ED discharge, 63% reported sustained headache relief for 48 h, while 37% continued to experience moderate to severe headaches. At the one-week follow-up, 53% reported no or rare headache occurrence.

An observational retrospective cohort single-center study at a US Army neurology clinic investigated treatment outcomes in 100 military personnel with *de novo* PTH attributed to mTBI [15]. Seventy-seven percent of the subjects had sustained a blast-related mTBI, while 23% had suffered a non-blast mTBI. In addition, 52% screened positive for post-traumatic stress disorder, while 38% screened positive for depression. More than 90% of the subjects presented with a migraine-like headache. The mean time since PTH-onset was 18 months, and the mean headache frequency was 17 days per month. This study mainly focused on preventive PTH treatment but did also provide data on acute PTH treatment. The author reported that triptans (*n* = 73 and a 70% responder rate) were more effective compared to non-triptans (*n* = 33 and a 42% responder rate) in terms of headache relief (not further specified) within two hours after intake (*P = 0.01*). Notably, 23% used more than one abortive medication.

### Preventive pharmacological treatment of post-traumatic headache

Three studies investigated preventive pharmacological PTH treatment. One study included military personnel [15], one study included a pediatric sample [14] and one study included a combined adult and pediatric population [13]. The list of preventive medications used included: amitriptyline [13–15], nortriptyline [14, 15], topiramate [14, 15], propranolol [15], valproate [15], melatonin [14], gabapentin [13] and flunarizine [14].

In the aforementioned military study [15], 100 subjects received the following prophylactic medications: tricyclic antidepressants (amitriptyline or nortriptyline, *n* = 48) 25–50 mg/day, topiramate (*n* = 29) 100 mg/day, propranolol (*n* = 18) 80 mg/day and valproate (*n* = 5) 500 mg/day). Some patients also received non-pharmacological treatment (e.g. physical therapy, neuropsychology) during the observational period. Subjects were allowed to use abortive medication such as NSAIDs (n = 18) and triptans (*n* = 73). Primary outcome measures were assessed by phone three months after baseline evaluation and included: 1) number of days in the previous month with a headache lasting more than 30 min, 2) headache-related disability as determined by Migraine Disability Assessment (MIDAS) questionnaire, and 3) whether headaches were reliably relieved within 2 h after intake of abortive medication. This study found that headache frequency decreases with 2.6 days (− 15.5%, *P = 0.009*) at follow-up compared to baseline after the subjects started on prophylactic treatment. Thirty-five percent experienced a ≥ 50% decrease in headache frequency. The decrease in headache frequency was significant for subjects treated with topiramate (*n* = 29, *P = 0.02*), but not for any of the other prescribed prophylactic treatments. At three months post-baseline, 66 of 100 subjects (34% medication discontinuation rate) were still taking the prophylactic treatment that was prescribed at baseline. Moreover, the study found a 57% decrease in headache-related disability among all subjects as measured by MIDAS. In conclusion, the authors stated that topiramate could be an effective prophylactic drug for PTH, while triptans appeared to be the most efficient abortive medication for PTH. However, these findings would need to be validated in prospective, placebo-controlled RCTs.

Kuczynski et al. [14] investigated the efficacy of prophylactic PTH treatment using a retrospective chart review in 44 children (mean age: 14), who attended a brain injury clinic due to PTH. The mean time since traumatic brain injury (TBI) was 7 months (range: 1–29 months) and 61% reported daily headaches. The list of prophylactic treatments used in the study included: amitriptyline (5 mg/day to 1 mg/kg/day), nortriptyline (dosage not specified), topiramate (12.5 to 200 mg/day), melatonin (3–10 mg/day) and flunarizine (dosage not specified). The primary outcome was a ≥ 50% reduction in headache frequency and whether prophylactic treatments were continued for 3 months after headache resolution and subsequently gradually discontinued. The authors reported that the overall treatment response was 64%. More specifically, melatonin and amitriptyline yielded a treatment success rate of 75% (9 of 12 subjects) and 72% (13 of 18 subjects), respectively. However, 39% received more than one treatment i.e. physical therapy and biofeedback therapy.

A retrospective cohort study in an academic sports medicine practice classified patients into three groups: gabapentin group (median dose: 900 mg, *n* = 60), amitriptyline group (median dose: 20 mg, *n* = 94) and no medication group (*n* = 123) [13]. The latter was defined as subjects who had not been prescribed any pharmacological treatment during the study period. No patient was prescribed both gabapentin and amitriptyline. A 7-point numeric rating scale from 0 to 6 (*0* no headache, *5–6* severe headache) was used to record headache scores. In the gabapentin and amitriptyline groups, most subjects (~ 80%) were prescribed pharmacological treatment at the first or second visits (median time since mTBI: 4.3 weeks) and inclusion was based on at least one follow-up visit after treatment initiation. Therefore, 146 subjects were excluded because they did not have follow-up measurements. The reasons for loss to follow-up were not available. For the 277 PTH patients that were included in the final analysis, the mean number of physician visits per patient was 4.1 during a mean period of 4.8 months. In both medication groups (gabapentin and amitriptyline), headache scores improved over time. However, headache scores improved similarly in the no medication group.

## Discussion

In conclusion, we found a lack of quality studies on both acute and preventive pharmacological treatment of PTH. Based on data reviewed from seven studies, efficacy of any pharmacological intervention could not be inferred as discussed below. The included studies used an open-label design limited by both investigator-subject interaction and placebo response. The latter varies widely across clinical trials with both acute and preventive medications for migraine [16–18]. A similar placebo effect could be expected in PTH patients. The primary and secondary endpoints varied between the included studies, making treatment comparisons unfeasible. In addition, 5 of 7 included studies included pediatric populations. In this context, it is worth noting that high placebo responses to pharmacological treatment have previously been reported in pediatric migraine patients [19]. Furthermore, the studies were also considerably underpowered with sample sizes ranging from 15 to 277 patients. In addition, the only prospective study included did not even establish baseline headache characteristics using a prospectively collected headache diary [11]. Some of the reviewed studies included subjects with substantial within-study variance in terms of time range from TBI to intervention [9, 10, 13–15]. As an example, the preventive treatment study [14] included 44 children with a mean time period since TBI of 7 months (range: 1–29 months). Thus, it is highly likely that some of the subjects experienced spontaneous reduction in headache frequency during the treatment period.

### Recommendations for future research

Further research in acute and preventive treatment for PTH is highly warranted. Data analysis can be greatly enhanced, if consensus guidelines for controlled trials of acute and preventive treatment of PTH are provided. We suggest several aspects that should be considered to improve data consistency and study design reliability. *First*, future RCTs should include a double-blinded, randomized, parallel-group study design. This would enable us to prove drug efficacy and provide robust information on safety and tolerability. *Second*, future open-label studies and RCTs should require included subjects to fulfill the ICHD criteria for PTH to reduce population heterogeneity. A future version of the ICHD may benefit from subdividing PTH based on headache phenotype, as there could be differences in treatment outcome depending on the headache phenotype. Along this line of reasoning, detailed characterization of initial injury severity and type (i.e. GCS or computer tomography/magnetic resonance imaging classification) would also offer much needed patient stratification, which in turn could provide more targeted treatment approaches for individual patients. *Third*, baseline data on headache characteristics (including headache phenotype), headache ‘triggers’ (i.e. physical and cognitive activity), comorbid psychiatric illness (e.g. anxiety and depression), number of TBIs, pre-trauma history of headache and other relevant data should be recorded prior to intervention. *Fourth*, primary and secondary endpoints should be similar among future studies. In this context, the International Headache Society (IHS) has provided guidelines for controlled trials of acute and preventive treatment of migraine [20, 21]. These guidelines could serve as inspiration, which would enable us to compare efficacy of acute and preventive migraine drugs between PTH and migraine patients. *Lastly*, PTH patients often present with a plethora of symptoms (i.e. fatigue, depression, sleep disturbances) and not only headache [2, 22]. It would be interesting if future RCTs and high-quality open-label studies included secondary endpoints set to investigate drug efficacy on factors such as health-related quality of life, work productivity and levels of anxiety and depression.

## Conclusion

This systematic review has shown a low level of evidence to support any pharmacological treatment of PTH. High-quality RCTs and open-label studies are needed to provide robust evidence of clinical utility. Future research efforts should be driven by rigorous methodology and improved outcome assessment of analytical validity. In addition, PTH patients display a myriad of comorbid symptoms (i.e. depression, anxiety, sleep disturbances). Thus, future studies should include multidimensional outcome scales that cover multiple symptom domains. This will facilitate more targeted treatment approaches that accounts for clinical variability between patients and better reflects the concept of precision medicine.

## Data Availability

The datasets used in the present review are available from the corresponding author on reasonable request.

## Abbreviations

ED: Emergency department
GON: Greater occipital nerve
ICHD: International Classification of Headache Disorders
MIDAS: Migraine Disability Assessment
MOH: Medication-overuse headache
mTBI: Mild traumatic brain injury
NRS: Numeric rating scale
NSAIDs: Nonsteroidal anti-inflammatory drugs
PTH: Post-traumatic headache
RCTs: Randomized clinical trials
TBI: Traumatic brain injury
